# ICTV Virus Taxonomy Profile: *Potyviridae*

**DOI:** 10.1099/jgv.0.000740

**Published:** 2017-04-01

**Authors:** Stephen J Wylie, Mike Adams, Celia Chalam, Jan Kreuze, Juan José López-Moya, Kazusato Ohshima, Shelly Praveen, Frank Rabenstein, Drake Stenger, Aiming Wang, F. Murilo Zerbini, ICTV Report Consortium

**Affiliations:** ^1^​ State Agricultural Biotechnology Centre, Murdoch University, Perth, Western Australia 6150, Australia; ^2^​ 24 Woodland Way, Stevenage, Herts SG2 8BT, UK; ^3^​ Division of Plant Quarantine, ICAR – National Bureau of Plant Genetic Resources, Pusa Campus, New Delhi 110012, India; ^4^​ International Potato Center (CIP), Apartado 1558, Lima 12, Peru; ^5^​ Centre for Research in Agricultural Genomics CRAG, CSIC-IRTA-UAB-UB, Campus Bellaterra, 08193 Barcelona, Spain; ^6^​ Laboratory of Plant Virology, Department of Applied Biological Sciences, Faculty of Agriculture, Saga University, 1-banchi, Honjo-machi, Saga 840-8502, Japan; ^7^​ Division of Biochemistry, Indian Agricultural Research Institute, New Delhi 110012, India; ^8^​ Institute for Epidemiological and Pathogen Diagnosis, Julius Kuhn Institut, Quedlinburg, Saxony-Anhalt, Germany; ^9^​ Crop Diseases, Pests, Genetics, ARS, USDA, 9611 S. Riverbend Ave., Parlier, CA 93648, USA; ^10^​ London Research and Development Centre, Agriculture and Agri-Food Canada, 1391 Sandford St., London, Ontario N5V 4T3, Canada; ^11^​ Dep. de Fitopatologia/BIOAGRO, Universidade Federal de Viçosa, Viçosa, MG, 36570-900, Brazil

**Keywords:** *Potyviridae*, ICTV Report, Taxonomy

## Abstract

The *Potyviridae* is the largest family of RNA plant viruses, members of which have single-stranded, positive-sense RNA genomes and flexuous filamentous particles 680–900 nm long and 11–20 nm wide. There are eight genera, distinguished by the host range, genomic features and phylogeny of the member viruses. Genomes range from 8.2 to 11.3 kb, with an average size of 9.7 kb. Most genomes are monopartite but those of members of the genus *Bymovirus* are bipartite. Some members cause serious disease epidemics in cultivated plants. This is a summary of the International Committee on Taxonomy of Viruses (ICTV) Report on the taxonomy of the *Potyviridae*, which is available at www.ictv.global/report/potyviridae.

## Virion

The flexuous, filamentous particles are 680–900 nm long and 11–20 nm wide, with helical symmetry and a pitch of about 3.4 nm (Table 1). Particles of some viruses are longer in the presence of divalent cations than in the presence of EDTA. Virion sedimentation coefficient S_20,w_ is 137–160S; density in CsCl is 1.31 g cm^−3^; extinction coefficient E^0.1 %^_1cm, 260nm_=2.4–2.7. Virions contain a core capsid protein (CP) of 30–47 kDa; the tip of the capsid may contain the virus-encoded proteins genome-linked protein (VPg) and helper-component proteinase (HC-Pro) [1]. Virions contain about 5 % RNA by weight [2]. Virions are moderately immunogenic; there are serological relationships among many members. Some monoclonal antibodies react with most aphid-transmitted potyviruses [3, 4].

**Table 1.** Characteristics of the family *Potyviridae*

Typical member:	potato virus Y-O (U09509), species *Potato virus Y*, genus *Potyvirus*
Virion	Non-enveloped, flexuous and filamentous capsid, 680–900 nm long and 11–20 nm in diameter with a single core capsid protein
Genome	8–11 kb of positive-sense, single-stranded, usually monopartite RNA (bipartite in the genus *Bymovirus*)
Replication	Cytoplasmic, initiated in virus replication complexes on membranous vesicles at ER exit sites. Replication initiates at 6K2-induced ER-originated vesicles
Translation	Directly from genomic RNA
Host range	Plants (all virus genera). Most members are arthropod-borne but those of the genus *Bymovirus* are transmitted by plasmodiophorids
Taxonomy	Currently eight genera containing nearly 200 species

## Genome

**Fig. 1.**
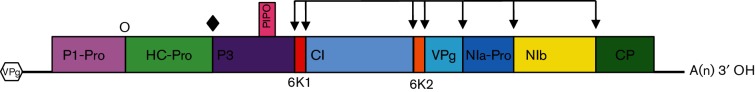
Genome organization of a typical member of the genus *Potyvirus*. Viruses of other genera may differ as described in Table 2. VPg, viral protein genome-linked; P1-Pro, protein 1 protease; HC-Pro, helper component protease; P3, protein 3; PIPO, pretty interesting *Potyviridae* ORF; 6K, six kilodalton peptide; CI, cytoplasmic inclusion; NIa-Pro, nuclear inclusion A protease; NIb, nuclear inclusion B RNA-dependent RNA polymerase; CP, coat protein. Cleavage sites of P1-Pro (**O**), HC-Pro (♦) and NIa-Pro (↓) are indicated.

The single-stranded, positive-sense genome ranges from 8.2 kb (members of the species *Artichoke latent virus*, genus *Macluravirus*) to 11.3 kb (members of the species *Wheat yellow mosaic virus*, genus *Bymovirus*), with an average of around 9.7 kb (Table 2). The genomes have a VPg covalently linked to the 5′ end, and the 3′ terminus is polyadenylated. Most genomes are monopartite but those of members of the genus *Bymovirus* are bipartite. The genome of members of the species *Potato virus Y*, the type species of the genus *Potyvirus*, is organized as described in Fig. 1. The single large ORF of monopartite genomes encodes a single polyprotein that is cleaved into functional proteins at semi-conserved sites by three self-encoded proteases. Bipartite *Bymovirus* genomes encode two polyproteins that are cleaved by two proteases [5]. A second small ORF, PIPO, is generated by a polymerase slippage mechanism and is expressed as the *trans*-frame protein P3N-PIPO [6–8]. Coding region order and protein sequences are generally conserved throughout the family, although one of or both the P1 and the HC-Pro N-terminal coding regions may be lacking and a genus-specific or species-specific region may be present instead (Table 2). The coat protein of most isolates of the type species, *Potato virus Y*, contains 267 amino acids.

**Table 2.** Characteristics of members of the eight genera and two unassigned species in the family *Potyviridae*

Genus	Type species	Genome organization	Genome size range (kb)	Host range	Vectors	Notable features (*Alk-1*, activin receptor-like kinase-1; HC-Pro, helper-component protease; NTR, non-translated region; P1, protein one; RNAi, RNA-interference)
*Brambyvirus* (1 species)	*Blackberry virus Y*	Monopartite	10.8	*Rubus* species	Unknown	*Alk1* domain encoded in a very large P1 coding region. HC-Pro lacks motifs for genome amplification and systemic movement.
*Bymovirus* (6 species)	*Barley yellow mosaic virus*	Bipartite	RNA1 : 7.2–7.6 RNA2 : 2.2–3.6	Gramineae	*Polymyxa graminis*	Members lack P1 and HC-Pro coding regions. RNA2 encodes an HC-Pro-like protein unique to bymoviruses.
*Ipomovirus* (6 species)	*Sweet potato mild mottle virus*	Monopartite	9.0–10.8	Wide	Whitefly (*Bemisia tabaci*)	Some members lack the P1 and/or HC-Pro coding regions and encode a P1b protein instead, performing a role in host RNAi suppression.
*Macluravirus* (8 species)	*Maclura mosaic virus*	Monopartite	8.2	Wide	Aphids	No P1 coding region and the HC-Pro region is shorter than in potyviruses.
*Poacevirus* (3 species)	*Triticum mosaic virus*	Monopartite	9.7–10.2	Gramineae and Orchidaceae	Wheat curl mite (TriMV)	Unusually long 5′ NTR with 12 translation initiation codons and three ORFs.
*Potyvirus* (160 species)	*Potato virus Y*	Monopartite	9.4–11.0	Wide	Aphids	
*Rymovirus* (3 species)	*Ryegrass mosaic virus*	Monopartite	9.4–9.5	Gramineae	Eriophyid mites	
*Tritimovirus* (6 species)	*Wheat streak mosaic virus*	Monopartite	9.2–9.6	Gramineae	Eriophyid mites	P1 protein rather than HC-Pro serves as a suppressor of gene silencing.
Unassigned	*Rose yellow mosaic virus*	Monopartite	9.5	*Rosa* sp.		
Unassigned	*Spartina mottle virus*	Unknown	Unknown	Gramineae	Unknown	

## Replication

Viruses are transmitted horizontally by arthropods or plasmodiophorids; some are transmitted vertically in seed. Some members cause serious disease epidemics in cultivated plants. Members of a few species infect over 30 plant families, but most infect one or a few host species or families [9] (Table 2).

## Taxonomy

The family is divided into eight genera, the members of which are distinguished by host range, genomic features and phylogeny (Table 2). The species demarcation criteria, based upon the large ORF or its protein product, are generally accepted as <76 % nucleotide identity and <82 % amino acid identity. If the complete ORF sequence is not available, similar criteria can be used for the coat protein coding region and its product. The corresponding thresholds for species demarcation using nucleotide identity values for other coding regions range from 58 % (P1 coding region) to 74–78 % (other regions), although these ranges are exceeded in some cases [10].

## Resources

Full ICTV Online (10th) Report: www.ictv.global/report/potyviridae.
